# The Metropolitan Hospital Sunday Fund

**Published:** 1907-08-10

**Authors:** 


### HOSPITAL ADMINISTRATION.

### CONSTRUCTION AND ECONOMICS.

### THE METROPOLITAN HOSPITAL SUNDAY FUND.

HOSPITAL EXTENSIONS: AN ATTEMPT TO CHECK EXTRAVAGANT BUILDING.

The meeting of the Hospital Sunday Fund Council at the Mansion House on Thursday, 1st inst., owing no doubt to the commencement of the holidays, was not largely attended. We mention this fact because the discussion fairly indicates the general feeling which prevails in London at the present time in regard to great projects for new hospital buildings, which it may be proposed shall be entered upon before the money to defray their cost, or the income to maintain them when erected, has been procured. The secretary mentioned several instances where large sums of money had been spent in new buildings before adequate funds were forthcoming, with the result that many hundreds of beds are still unoccupied, and the new buildings are gradually deteriorating, because they have to remain empty for want of funds. The mere statement of facts like these is sufficient to justify action on the part of the central funds, whose function it should be not only to enforce economical administration, but to check hasty expenditure upon new buildings. Alderman Bell had given notice of a resolution in the following terms: "That before any hospital or institution receiving a grant from this fund enters into a contract for the provision of additional accommodation for patients, or any extraordinary expenditure which would increase the cost of maintaining the hospital, the consent or approval of the council of this Fund must be obtained."

Anyone who considers the matter fairly must agree that the wording of this resolution was not happy, and that the effect of it, if passed, must have been to bring the Hospital Sunday Fund into direct conflict with the managers of the principal hospitals in London. Sir Henry Burdett and others pointed this out, and the former suggested amendments which made the resolution take the form of a notice to be given to each institution seeking a grant, that the Distribution Committee had been instructed to provide that each participating institution should submit, for the approval of the Council, any proposals which it might have in hand for additional accommodation for patients, or any extraordinary expenditure. In this modified form the resolution was less drastic, but the general feeling of the meeting was in favour, as a preliminary step, of first inviting the hospitals to submit their proposals for the provision of additional accommodation for patients, and to omit for the present any request as to extraordinary expenditure. In this modified form the Council unanimously decided to agree to support the principle of the proposal embodied in Alderman Bell's resolution.

There can be no reasonable doubt that the success which has attended the efforts of the three great funds to raise money in recent years has tended to increase the demands of the medical staff of several hospitals in favour of exceedingly costly plant and buildings for new departments of hospital work, and in addition the elaboration of old buildings in certain directions—*i.e.*, the provision of far too expensive operating theatres—to an extent which, if continued, must tend to discourage the voluntary system of hospital support, if it does not destroy it altogether. It is time, having regard to the existing depression, to the ever-increasing taxes, to the heavy burden of the present income-tax, which presses especially hardly upon those very members of the community from whom the large majority of hospital subscribers are drawn, that a halt should be called in regard to the provision of additional building accommodation for patients in the London hospitals. We have said more than once, and we believe from inquiries we have made, that the existing accommodation is ample to supply all the needs of the population of London, if the beds are restricted to deserving cases only, upon a system which will give to residents in the Metropolis priority of claim upon every free bed. At the present time the tendency in some hospitals is to admit cases sent from the country, where there may be very often adequate hospital accommodation, to the exclusion of patients residing in the immediate neighbourhood of a Metropolitan hospital. Wherever such a system has grown up, owing to the plan whereby the members of the honorary staff have a right to give priority of admission to patients who are sent in by members of the medical profession, the existing regulations should be most carefully overhauled by the Committees of Management, with a view to the removal of any abuses or injustices which may result therefrom to poor residents within the Metropolitan area. This change of system and stricter administration would, we believe, at once make some two thousand beds, if necessary, available for London cases. If anything like this number can be freed in this way, there can be no doubt that the great central funds will be more than justified in using their influence to restrict, with a firm and resolute hand, the provision of additional building accommodation for patients. In any case, the discussion at the Mansion House on the 1st inst. should have a very useful effect in many directions, and we hope it may have a tendency to enable some of the unoccupied beds in existing new buildings to be brought into use by strengthening the appeal for funds on the part of the preachers, and so increasing the revenue through the Hospital Sunday Fund, which will then be available for the upkeep of the London hospitals.

Mr. Pearce Gould was anxious that the Sunday Fund Council should invite everybody who proposed to organise a new hospital in any part of London to consult the Council before taking any step in that direction. The constitution of the Fund provides that no new hospital is eligible for a grant until it has published, and is able to present, the accounts for three complete years, so that, however desirable it may be to bring all proposals for the erection of additional hospitals to the test of knowledge and experience, it does not appear likely that the Hospital Sunday Fund alone could effectually exercise much control in the direction desired by Mr. Pearce Gould. Be this as it may, the point is a useful one, well worthy of full discussion. We are quite sure that the managers of the best administered hospitals throughout London will welcome the evidence which the discussion has afforded of the general interest which the public are exhibiting in the prevention of hasty and large expenditure upon additional hospital buildings in the circumstances mentioned.

